# H_2_O_2_ Mediates VEGF- and Flow-Induced Dilations of Coronary Arterioles in Early Type 1 Diabetes: Role of Vascular Arginase and PI3K-Linked eNOS Uncoupling

**DOI:** 10.3390/ijms24010489

**Published:** 2022-12-28

**Authors:** Naris Thengchaisri, Lih Kuo, Travis W. Hein

**Affiliations:** 1 Department of Medical Physiology, Cardiovascular Research Institute, School of Medicine, Texas A&M University Health Science Center, Bryan, TX 77807, USA; 2 Department of Companion Animal Clinical Sciences, Faculty of Veterinary Medicine, Kasetsart University, Bangkok 10900, Thailand

**Keywords:** coronary microvascular disease, endothelial dysfunction, nitric oxide, oxidative stress

## Abstract

In diabetes, the enzyme arginase is upregulated, which may compete with endothelial nitric oxide (NO) synthase (eNOS) for their common substrate L-arginine and compromise NO-mediated vasodilation. However, this eNOS uncoupling can lead to superoxide production and possibly vasodilator hydrogen peroxide (H_2_O_2_) formation to compensate for NO deficiency. This hypothesis was tested in coronary arterioles isolated from pigs with 2-week diabetes after streptozocin injection. The NO-mediated vasodilation induced by flow and VEGF was abolished by NOS inhibitor L-NAME and phosphoinositide 3-kinase (PI3K) inhibitor wortmannin but was not affected by arginase inhibitor N^ω^-hydroxy-nor-L-arginine (nor-NOHA) or H_2_O_2_ scavenger catalase in control pigs. With diabetes, this vasodilation was partially blunted, and the remaining vasodilation was abolished by catalase and wortmannin. Administration of L-arginine or nor-NOHA restored flow-induced vasodilation in an L-NAME sensitive manner. Diabetes did not alter vascular superoxide dismutase 1, catalase, and glutathione peroxidase mRNA levels. This study demonstrates that endothelium-dependent NO-mediated coronary arteriolar dilation is partially compromised in early type 1 diabetes by reducing eNOS substrate L-arginine via arginase activation. It appears that upregulated arginase contributes to endothelial NO deficiency in early diabetes, but production of H_2_O_2_ during PI3K-linked eNOS uncoupling likely compensates for and masks this disturbance.

## 1. Introduction

Diabetes mellitus is a common metabolic disease characterized by persistently high blood sugar levels resulting from a defect in insulin production (type 1) or a deficiency in insulin production/utilization (type 2). It is accompanied by a reduction in coronary perfusion and the dysregulation of coronary blood flow and vasomotor activity [1,2,3,4], especially with the presence of endothelial dysfunction in the microcirculation [3,5,6,7,8,9,10]. Shear stress, derived from the frictional force exerted on vascular endothelial cells by flowing blood, is essential for vascular homeostasis and a crucial determinant of vasomotor tone, vascular remodeling, and the development of thrombosis and atherosclerosis [11]. The ability of coronary microvessels to respond to increased shear stress, named flow-induced vasodilation [12], via the endothelial release of nitric oxide (NO) contributes to the regulation of coronary blood flow to match myocardial oxygen demand during metabolic activation [13,14,15]. The impairment of flow-induced dilation in the coronary vascular resistance arterioles has been recognized in humans [16,17] and animals [6,18] with diabetes. However, the cellular mechanism underlying the coronary vasomotor dysfunction evoked by diabetes remains incompletely understood. It has been shown that vascular endothelial growth factor (VEGF) exerts ligand-dependent vasodilation via VEGF receptor 2 (VEGFR2) activation and stimulation of NO production from endothelial NO synthase (eNOS) [19,20]. Various vascular complications have been linked to VEGF signaling pathways, including coronary artery disease, retinopathy, nephropathy, neuropathy, and peripheral vascular diseases in diabetic patients [21,22]. On the other hand, shear stress also activates VEGFR2 to exert ligand-independent vasodilation for the flow regulation [20,23]. VEGFR2 activation stimulates Akt phosphorylation and the subsequent regulation of eNOS activity plays a pivotal role in maintaining NO homeostasis [20,24].

Compromised endothelial function and reduced NO bioavailability are generally associated with diabetic vascular complications. The eNOS function can be altered by several diseases, including diabetes, to produce superoxide instead of NO [25,26]. This alteration is referred to as “eNOS uncoupling” [25,27,28,29]. Interestingly, it has been shown that hydrogen peroxide (H_2_O_2_) is formed from superoxide via superoxide dismutase and can modulate various endothelial functions in health and disease [30,31]. H_2_O_2_ is a potent vasodilator in coronary arterioles [32] and has been implicated as an endothelium-derived hyperpolarizing factor in many organs, including human and porcine coronary circulations [33]. However, it is unclear whether the reactive oxygen species generated by the uncoupled eNOS and the production of the vasodilator H_2_O_2_ can play a role in compensating for or modulating endothelium-dependent NO-mediated dilation of coronary arterioles. Although VEGFR2 can participate in diabetes-induced vascular complications, the impact of diabetes on vasomotor function mediated by VEGFR2 activation via ligand-dependent (i.e., VEGF) and -independent (i.e., shear stress) mechanisms has not been evaluated. Moreover, it has been implicated that upregulation of arginase, an L-arginine-consuming enzyme, can lead to eNOS uncoupling under vascular stress [34,35,36,37]. However, the role of arginase in eNOS uncoupling and H_2_O_2_ signaling in the participation of vasomotor dysregulation under early diabetic insult has not been investigated.

Because the development of microvascular complications is commonly associated with diabetes [38] and coronary artery disease is a major cause of death in diabetes [39,40], understanding the mechanism of coronary microvascular dysfunction elicited by early diabetes is important for designing effective therapy to treat coronary blood flow dysregulation and prevent organ damage. In the present study, we investigated the interaction between eNOS, arginase, NO, and H_2_O_2_ in modulating endothelium-dependent dilations of coronary arterioles in response to increased flow and VEGF in pigs 2 weeks after the induction of type 1 diabetes by streptozocin injection. We tested the hypothesis that the formation of vasodilator H_2_O_2_, due to eNOS uncoupling, compensates for the endothelial NO deficiency for coronary arteriolar dilation in the early stages of diabetes. The vasomotor activity was evaluated using an isolated and pressurized vessel approach to eliminate confounding influences from changes in hemodynamic and humoral factors that cannot be excluded under in vivo preparations.

## 2. Results

### 2.1. Early Diabetes Impairs Flow- and VEGF-Induced Vasodilations

**Figure 1:**
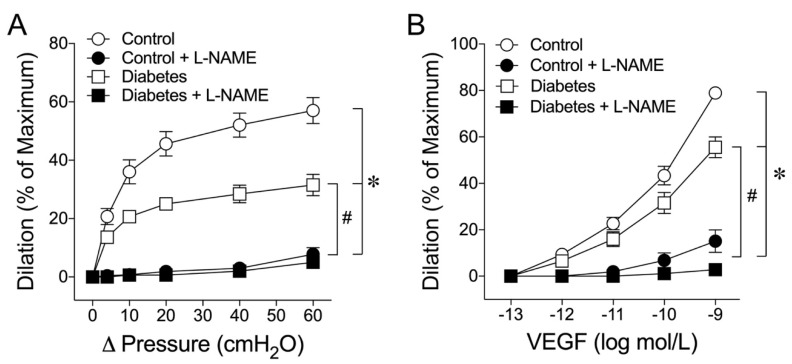
The role of NO in mediating flow- and VEGF-induced dilations was investigated in coronary arterioles isolated from control and diabetic pigs. (**A**): The elevated luminal flow, driven by the longitudinal pressure gradient (Δ pressure) across the vessel, caused vasodilation in normal pigs (n = 9). This vasodilation was compromised in the vessels isolated from diabetic pigs (n = 6). Administration of NOS inhibitor L-NAME (10 µmol/L) abolished the flow-induced vasodilation in both the control (n = 9) and diabetic pigs (n = 6). (**B**): Coronary arterioles from the control pigs (n = 7) dilated to VEGF in a concentration-dependent manner. VEGF-induced vasodilation was compromised by diabetes (n = 5). Administration of L-NAME abolished the vasodilation in both the control (n = 7) and diabetic (n = 5) vessels. The resting vascular tones were not altered before and after L-NAME in either control (63 ± 1% vs. 62 ± 1%) or diabetic (55 ± 3% vs. 58 ± 4%) vessels. * *p* < 0.05 vs. control, # *p* < 0.05 vs. diabetes.

In the present study, the average maximum internal diameters for the arterioles from the control and diabetic pigs were 84 ± 2 µm and 79 ± 3 µm, respectively. The vessels were pressurized to 60 cmH_2_O hydrostatic pressure without flow by two independent reservoirs [12]. The reason for selecting this luminal pressure was based on the in vivo coronary pressure measurements of about 40–50 mmHg in this size of coronary arterioles [41]. The pressurized control vessels developed spontaneous basal tone about 60% to 65% of the maximum diameter within 30–40 min under a 36–37 °C bath temperature. The vessels from the diabetic pigs developed a higher level of basal tone about 50% to 55% of the maximal diameter. The luminal flow of the vessel was initiated by moving the pressure reservoirs in an opposite direction to create a pressure gradient (Δ pressure) across the vessel longitudinally without changing the luminal pressure as described previously [12]. This dual-reservoir system eliminated the confounding effect of the pressure-dependent myogenic response during changes in the luminal flow [14]. The control vessels dilated to elevated flow in a manner dependent upon the magnitude of the pressure gradient, and administration of NOS inhibitor N^ω^-nitro-L-arginine methyl ester (L-NAME, 10 µmol/L) completely blocked this flow-induced vasodilation (Figure 1A). The vessels isolated from diabetic animals also exhibited significant vasodilation to the elevated flow, but the magnitude was compromised compared with that of the control animals (Figure 1A). Administration of L-NAME also completely abolished the flow-induced dilation in diabetic vessels (Figure 1A). Administration of VEGF caused concentration-dependent dilations of both the control and diabetic vessels in a manner sensitive to L-NAME. However, the diabetic vessels were less responsive to VEGF (Figure 1B).

### 2.2. Role of H_2_O_2_ in Flow- and VEGF-Induced Coronary Arteriolar Dilations

**Figure 2:**
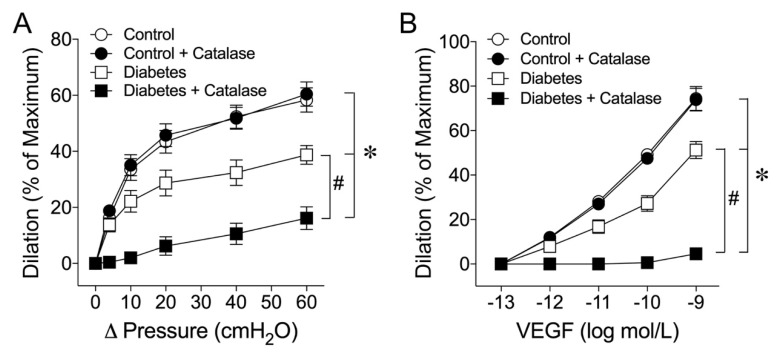
The role of H_2_O_2_ in mediating flow- and VEGF-induced dilations was investigated in coronary arterioles isolated from control and diabetic pigs. (**A**): The vasodilation induced by a stepwise increase in pressure gradient (Δ pressure), i.e., flow, in control pigs (n = 6) was not affected by H_2_O_2_ scavenger catalase (1000 units/mL; n = 6). The blunted flow-induced dilation in diabetic vessels (n = 8) was further inhibited by catalase (n = 8). (**B**): The vasodilation induced by VEGF was not affected by catalase in the control pigs (n = 5). In diabetic pigs (n = 6), the vasodilation to VEGF was blunted and the remaining dilation was abolished by catalase. The resting vascular tones were not altered before and after catalase in either the control (61 ± 2% vs. 62 ± 2%) or diabetic (60 ± 2% vs. 60 ± 2%) vessels. * *p* < 0.05 vs. control, # *p* < 0.05 vs. diabetes.

To address whether H_2_O_2_ contributes to the flow- and VEGF-induced vasomotor responses, the vessels were treated with H_2_O_2_ scavenger catalase (1000 units/mL). Catalase did not affect the flow-induced dilation in the control vessels. However, in the vessels from the diabetic animals, the vasodilation in response to increased flow was compromised and the addition of catalase further blunted the vasodilation (Figure 2A). Similarly, VEGF-induced dilation was compromised in diabetic vessels. Administration of catalase did not affect VEGF-induced dilation in the control vessels but abolished the vasodilator response in the diabetic vessels (Figure 2B).

### 2.3. Coronary Arteriolar Dilations to NO Donor and H_2_O_2_

**Figure 3:**
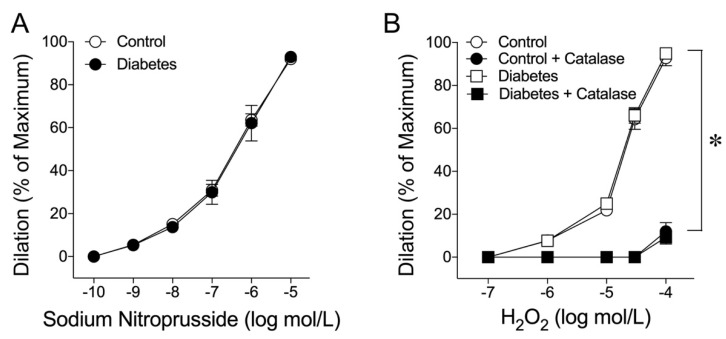
Responses of coronary arterioles to NO donor sodium nitroprusside and H_2_O_2_. (**A**) Both the control (n = 5) and diabetic (n = 5) vessels dilated to sodium nitroprusside in a concentration-dependent manner without differences. (**B**) H_2_O_2_ produced similar dilations in the control (n = 5) and diabetic (n = 6) vessels and catalase (1000 units/mL) abolished the dilations in the control (n = 5) and diabetic (n = 6) vessels in the same manner. * *p* < 0.05, control/diabetes vs. catalase treatment.

It is unclear whether early diabetes affects the response of the blood vessel to the NO donor or reactive oxygen species H_2_O_2_. To address this issue, the dilations of coronary arterioles to NO donor sodium nitroprusside and H_2_O_2_ were examined and compared in vessels isolated from the control and diabetic pigs. Administration of sodium nitroprusside caused similar concentration-dependent dilations of both control and diabetic vessels (Figure 3A). Coronary arterioles also dilated in response to H_2_O_2_ in a similar manner in both the control and diabetic vessels, and this vasodilation was abolished by catalase (1000 units/mL), except for the highest concentration of H_2_O_2_ (Figure 3B).

### 2.4. Vascular L-Arginine Availability and the Role of Arginase in Early Diabetes

**Figure 4:**
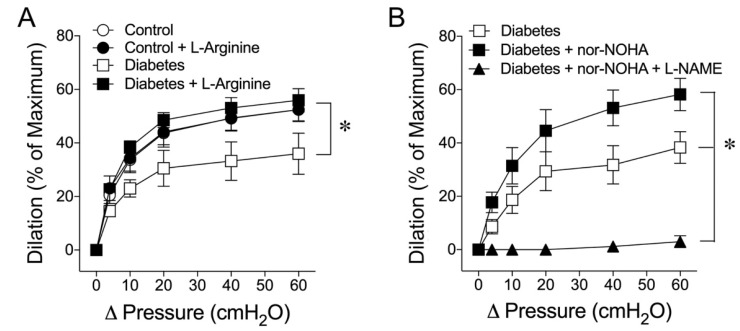
The impacts of L-arginine availability and arginase activity on flow-induced dilation were evaluated in coronary arterioles from pigs with and without diabetes. (**A**) Administration of L-arginine (3 mmol/L; n = 6) did not alter flow-induced dilation in the control vessels (n = 6) but the impaired vasodilation by diabetes (n = 5) was restored by L-arginine (n = 5). (**B**) The blunted flow-induced dilation in diabetic vessels (n = 6) was reversed by arginase inhibitor nor-NOHA (0.1 mmol/L; n = 6). Co-incubation with nor-NOHA and L-NAME abolished the flow-induced dilation of coronary arterioles in diabetic pigs (n = 4). The resting vascular tones were not altered before and after L-arginine in either control (59 ± 4% vs. 58 ± 3%) or diabetic (62 ± 3% vs. 63 ± 4%) vessels. nor-NOHA also did not alter the resting tone in diabetic vessels (62 ± 3% vs. 62 ± 4%). * *p* < 0.05 vs. diabetes.

The impacts of early diabetes on L-arginine availability and arginase activity for flow-induced dilation were investigated in coronary arterioles isolated from pigs with and without diabetes. Administration of L-arginine (3 mmol/L) to the control vessels did not affect flow-induced dilation. However, the vasodilation that was blunted by diabetes showed significant improvement by L-arginine, and the vasodilation magnitude was restored to the control level (Figure 4A). Furthermore, treating the diabetic vessels with arginase inhibitor N^ω^-hydroxy-nor-L-arginine (nor-NOHA; 0.1 mmol/L) restored flow-induced dilation, in a manner sensitive to L-NAME (Figure 4B).

### 2.5. Role of Phosphoinositide 3-Kinase (PI3K) in Flow- and VEGF-Induced Vasodilations

**Figure 5:**
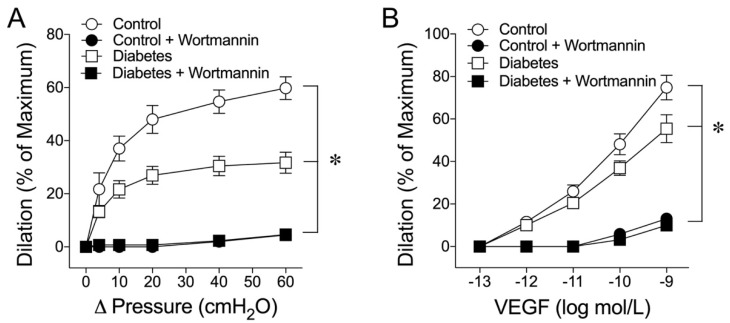
Effect of PI3K inhibition on flow- and VEGF-induced coronary arteriolar dilations. (**A**) The dilation of coronary arterioles to increased luminal flow (Control; n = 4) was attenuated by diabetes (n = 4) and administration of PI3K inhibitor wortmannin (0.1 µmol/L) abolished dilation of the control (n = 5) and diabetic (n = 5) vessels. (**B**) The VEGF-induced vasodilation (Control; n = 5) was abolished by wortmannin (n = 5) and the dilation of the diabetic vessels (n = 5) was also abolished by wortmannin (n = 5). The resting vascular tones were not altered before and after wortmannin in either control (64 ± 2% vs. 64 ± 2%) or diabetic (62 ± 3% vs. 65 ± 2%) vessels. * *p* < 0.05 vs. diabetes.

Activation of PI3K can lead to eNOS activation and NO production. However, it is unclear whether this signaling pathway contributes to the dilations of coronary arterioles elicited by flow and VEGF. It is also unclear whether this pathway is compromised in early diabetes. Therefore, the involvement of PI3K in these vasomotor responses was investigated and compared in coronary arterioles isolated from the control and diabetic pigs. Administration of PI3K inhibitor wortmannin (0.1 µmol/L) did not alter resting vascular tone but eliminated dilations in response to elevated flow (Figure 5A) and VEGF (Figure 5B) in both the control and diabetic vessels.

### 2.6. Role of Vascular Superoxide Dismutase 1 (SOD1), Catalase, and Glutathione Peroxidase (GPX) mRNA Expressions

**Figure 6:**
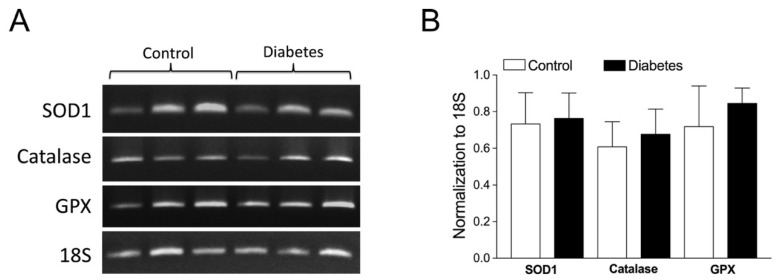
Effect of early diabetes on the mRNA expressions of SOD1, catalase, and GPX in isolated coronary arterioles. (**A**) The expressions of antioxidant genes (SOD1, catalase, and GPX) were determined by RT-PCR and the expressions were normalized to the 18S transcripts. (**B**) The quantitative analysis showed no difference in the antioxidant gene expressions between the control (n = 3) and diabetic (n = 3) coronary arterioles.

To assess whether vascular antioxidants are altered by early diabetes, the mRNA expressions of SOD1, catalase, and GPX were evaluated in coronary arterioles isolated from pigs with and without early diabetes (Figure 6A). The quantitative analysis of mRNA expression showed that the mRNA expressions of these antioxidant genes were comparable between the control and diabetic vessels (Figure 6B).

## 3. Discussion

The present study showed that the endothelium-dependent coronary arteriolar dilations to elevated flow and VEGF are impaired in the early stages (2 weeks of hyperglycemia) of type 1 diabetes in pigs, a large animal model resembling human cardiovascular physiology and pathophysiology. The compromised NO-mediated vasodilation can be restored by the supplementation of eNOS substrate L-arginine or by the inhibition of arginase activity. The released H_2_O_2_ during both flow and VEGF stimulations compensates for the NO deficiency and maintains, at least in part, the endothelial function. Early diabetes is likely to increase coronary arteriolar arginase activity leading to eNOS uncoupling and consequently promotes the formation of H_2_O_2_ to substitute for NO to mediate arteriolar dilation to increased flow and to VEGF.

We have previously shown that NO-dependent coronary arteriolar dilations in response to pharmacological stimulation by serotonin are impaired in pigs subjected to 2 weeks of type 1 diabetes [10]. However, it remains to be determined whether the physiological stimulation with the elevated flow in the coronary microcirculation is affected by early diabetes. This question is important because this vasomotor response is expected to play a critical role in regulating coronary blood flow during metabolic activation [12,13,42,43,44]. Our results showed that coronary arteriolar dilation to increased flow is partially compromised in the early stages of diabetes (Figure 1A). This finding agrees with the reduced flow-induced dilation in the brachial artery as one of the early changes in cardiovascular parameters in human patients with type 1 diabetes [45], although it is unclear whether this impairment extends to the microcirculation. In human patients with type 1 diabetes, the increased flow elicited by the endothelium-dependent vasodilator acetylcholine or by a physiological stimulation following a sudden release of arterial occlusion (i.e., reactive hyperemia) was compromised in the skin microcirculation [46]. Moreover, the coronary blood flow reserve, which is known to be directly related to the ability of coronary microvessels to dilate, was reportedly reduced in young patients with type 1 diabetes without microalbuminuria and autonomic neuropathy [47] or any clinical cardiovascular abnormalities [48]. Interestingly, the adenosine-induced increase in coronary blood flow was compromised in young men with uncomplicated type 1 diabetes [49]. These clinical studies may suggest the development of coronary arteriolar dysfunction related to NO deficiency because it has been shown that adenosine stimulates the endothelial release of NO to exert dilation in small coronary arterioles [50,51]. Because endothelial NO contributes significantly to the capacity of coronary flow reserve [52] and vasodilation elicited by shear stress (Figure 1), thus linking to metabolic activation [43], the negative impact of early diabetes on blood flow regulation in the coronary microcirculation is apparent.

Myocardial perfusion is optimally regulated to match the metabolic activity of the myocardium by adjusting microvascular resistance located at the size of arterioles less than 150 µm in diameter [53]. In the presence of NOS blockade, administration of catalase to the intact coronary circulation causes a substantial reduction in coronary arteriolar dilation in response to pacing-induced increases in myocardial oxygen consumption [54], indicating the important role of endogenous H_2_O_2_ in mediating coronary arteriolar dilation in vivo. However, the specific cellular source of H_2_O_2_ cannot be identified based on the in vivo experiments alone because of the complexity of coronary blood flow regulation and the disturbance of multiple confounding factors that cannot be separated in the intact animal preparations. Moreover, the mechanism of coupling H_2_O_2_ production to vasomotor regulation in coronary arterioles has not been established. Combining both in vivo and in vitro approaches, it was demonstrated that H_2_O_2_ released from the cardiomyocytes couples myocardial oxygen consumption to coronary blood flow via a feed-forward mechanism [55]. Interestingly, H_2_O_2_ predominantly acts on small coronary arterioles less than 100 µm in diameter [54], the size of vessels that is consistent with the current study. Furthermore, H_2_O_2_ has been shown to exert a cardioprotective role in coronary ischemia-reperfusion injury by compensating for impaired NO-mediated dilation in vivo [56]. However, the underlying mechanism is unclear and whether coronary arterioles are the source of H_2_O_2_ in the disease state has not been determined. In the present study, we found that the NO-mediated dilations in small coronary arterioles (~50 µm with basal tone) in response to flow and VEGF were compromised by about 40% in early diabetes. However, H_2_O_2_ appears to play a compensatory role in maintaining the remaining dilations of diseased vessels because catalase nearly abolished these responses only in vessels isolated from the diabetic animals (Figure 2). Because the isolated vessels were free from cardiomyocytes, this compensatory response appears to be derived from the blood vessel per se. It is worth noting that the vasodilation in response to the NO donor sodium nitroprusside was not altered by diabetes (Figure 3A), suggesting that the compromised vasodilation was not related to the alteration of smooth muscle NO signaling. In isolated coronary arterioles, the dilation to H_2_O_2_ was found to be mediated by the activation of endothelium-dependent signaling through the cycloxygenase-1-mediated release of prostaglandin E2 and the direct relaxation of smooth muscle through the opening of calcium-activated potassium channels [32]. These endothelium-dependent and -independent signaling pathways appear to be intact in early diabetes because vasodilation induced by H_2_O_2_ was not affected (Figure 3B).

The alteration in eNOS function might be the culprit in the present study because both flow- and VEGF-induced dilations were sensitive to eNOS blockade in both healthy and diabetic vessels (Figure 1). Because of the involvement of H_2_O_2_ in mediating dilations in diabetic vessels (Figure 2) in a manner sensitive to L-NAME (Figure 1), it is likely that the H_2_O_2_-mediated compensatory response originated from the eNOS signaling in the diseased vessel. This context was supported by the restoration of NO-mediated dilations by a high concentration of eNOS substrate L-arginine in the diabetic vessels (Figure 4A). The deficiency of L-arginine might have caused the uncoupling of eNOS for superoxide production and the subsequent formation of H_2_O_2_ [25,26,27,28,29] via superoxide dismutase in diabetic vessels. In addition, the increased activity of L-arginine-consuming enzyme arginase might have contributed to the eNOS uncoupling by reducing L-arginine bioavailability because the impaired vasodilation can be restored by the arginase inhibitor nor-NOHA (Figure 4B). Although arginase is constitutively expressed in the coronary vasculature [57], it can be upregulated and consequently contribute to endothelial NO deficiency and vascular dysfunction in various forms of vascular diseases and medical conditions such as aging [58], ischemia-reperfusion injury [34], systemic hypertension [36], hemorrhagic shock [59], and diabetes mellitus [37] by initiating or following oxidative stress and inflammation associated with L-arginine deficiency [60]. However, in the present study, arginase activity was not assessed, because of insufficient microvascular protein samples available for these experiments. This limitation may be addressed if a high-sensitivity assay kit is available in the future.

A previous report, using the same diabetic pig model as in the present study, showed that sequential activation of lectin-like oxidized low-density lipoprotein receptor-1, c-Jun N-terminal kinase, and arginase elicits superoxide-dependent oxidative stress with impaired endothelial NO-mediated coronary arteriolar dilation to serotonin [10]. However, the source of the oxidant is unclear and the administration of catalase in the previous study did not affect the diabetes-induced coronary endothelial dysfunction in response to serotonin [10]. Interestingly, in the same diabetic pig model, the impaired endothelium-dependent NO-mediated dilations of retinal arterioles to bradykinin and histamine are not affected by L-NAME [61]. However, treating diabetic retinal vessels with nor-NOHA, but not superoxide dismutase mimetic TEMPOL, preserves both histamine- and bradykinin-induced dilations in an L-NAME-sensitive manner. These studies suggest the crucial role of arginase activation in impairing endothelial NO function that can be associated with or dissociated from oxidative stress, depending upon the tissue (i.e., coronary vs. retinal arterioles) or the pharmacological agent. Moreover, those previous studies do not suggest a role of H_2_O_2_ in compensating for the NO deficiency, although the contribution of arginase activation to endothelial dysfunction is evident [10,61]. The reason for these inconsistent results on the H_2_O_2_ contribution is unclear, but they may be related to the different mechanisms of eNOS activation between pharmacological vs. mechanical (shear stress) stimulations. Serotonin, histamine, and bradykinin are known to activate eNOS through phosphorylation of the Ser-1179/1177 site [62,63] and activation of the enzyme through dephosphorylation at Thr-497 [64]. Interestingly, both shear stress and VEGF phosphorylate eNOS at Ser-635 with a slower activation of the enzyme compared to the phosphorylation of Ser-1179 [65]. This is consistent with our observations that both shear stress and VEGF took about 2–3 min to dilate the vessel in contrast to the vasodilation induced by serotonin, histamine, and bradykinin within 30 s of applying the agent [10,61]. It is worth noting that catalase did not affect resting vascular tone in either the control or diabetic vessels and the role of H_2_O_2_ was only observed under eNOS activations by shear stress and VEGF in the diabetic vessels (Figure 2). It is likely that the different sites of signaling in uncoupled eNOS might contribute to the generation of superoxide and the formation of H_2_O_2_ in response to shear stress and VEGF stimulations.

The context of the compensatory role of H_2_O_2_ generation from eNOS uncoupling can be supported by the blocking effect of PI3K inhibitor wortmannin on vasodilations to VEGF and shear stress (Figure 5). In cultured endothelial cells, PI3K is phosphorylated and activated by VEGFR2 following shear stress [66] or VEGF [67] stimulations. In the intact blood vessels, we found that VEGF and luminal flow/shear stress can activate VEGFR2 for vasodilation via ligand-dependent and ligand-independent mechanisms, respectively [20]. It appears that these two stimulation pathways converge to eNOS via PI3K signaling for NO release because inhibition of PI3K by wortmannin abolished both dilations in the control and diabetic vessels (Figure 5) similar to the blockade of eNOS by L-NAME (Figure 1). Our findings in the intact coronary arterioles are consistent with previous studies showing that wortmannin blocks NO production from cultured endothelial cells stimulated by shear stress [23] or VEGF [68]. Therefore, blocking upstream eNOS signaling by wortmannin is expected to eliminate the component of H_2_O_2_ in vasodilations to VEGF and shear stress in a similar fashion as that exerted by L-NAME (Figure 1) and catalase (Figure 2) if the uncoupled eNOS is the source of H_2_O_2_. Interestingly, in cultured pulmonary artery endothelial cells, H_2_O_2_ has been shown to activate eNOS for NO production, and overexpression of catalase can significantly attenuate the shear stress-induced increase in H_2_O_2_, eNOS activation, and NO generation [69]. However, under our experimental settings, the antioxidant gene expressions, including catalase, were unaltered in diabetic coronary arterioles (Figure 6). Moreover, endothelial NO does not participate in the dilation of porcine coronary arterioles to H_2_O_2_ [32]. Therefore, the increase in NO production by eNOS, due secondarily to H_2_O_2_ formation, is unlikely to be involved in the present study, at least in the early stages of diabetes/hyperglycemia.

**Figure 7:**
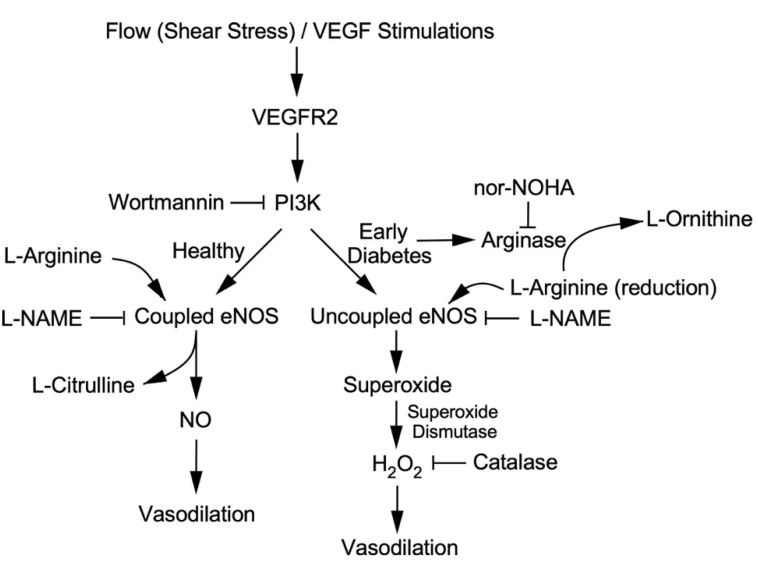
The diagram summarizes the pathways involved in the compensatory generation of H_2_O_2_ for NO deficiency in coronary arterioles due to arginase activation in the early stages of type 1 diabetes. L-Arginine is used by eNOS and arginase for the synthesis of NO and L-ornithine, respectively. In healthy coronary arterioles, shear stress and VEGF elicit endothelium-dependent NO-mediated vasodilation via activation of VEGR2/PI3K signaling. In the early stages of diabetes, arginase is activated and competes with eNOS for L-arginine. The reduction in L-arginine availability compromises the production of NO but causes PI3K-linked eNOS uncoupling and consequently promotes superoxide production and H_2_O_2_ formation via superoxide dismutase. The compromised NO-mediated vasodilation in early diabetes is compensated by H_2_O_2_. The inhibitors used for probing the signaling pathway are indicated.

In the present study, we conclude that mild oxidative stress, i.e., H_2_O_2_ formation during PI3K-linked eNOS uncoupling, corresponding to arginase activation and L-arginine deficiency, might be beneficial in terms of compensating for the NO deficiency to maintain the vasomotor function in the coronary microcirculation in early diabetes (Figure 7). However, this compensatory mechanism is not sufficient to fully restore the reduced endothelium-dependent NO-mediated function. This notion is consistent with the finding that the H_2_O_2_ released from the collateral microvessels improves the vasomotor function with exercise training in the ischemic heart [30]. On the contrary, prolonged hyperglycemia may exacerbate redox dysregulation in the vasculature, thereby providing positive feedback for developing adverse diabetic complications [70]. It is worth noting that the excessive production and prolonged exposure of microvessels to H_2_O_2_ can severely impair NO-mediated endothelial function by further reducing L-arginine availability through hydroxyl radical-dependent upregulation of arginase [35]. Therefore, redox signaling can be a double-edged sword in the microcirculation, which may help tissue survival during early disease stress by compensating for vasomotor regulation in one way and eliciting uncontrolled global oxidative stress and irreversible vascular damage for tissue injury in another way in the advanced stages of disease development. The early treatment of microvascular dysfunction by arginase inhibition might be a worthwhile strategy to consider for preventing the progression of microvascular complications toward severe tissue injury.

## 4. Materials and Methods

### 4.1. Porcine Diabetes Model

The approval of animal procedures was obtained from the Texas A&M University Health Science Center/Baylor Scott and White Institutional Animal Care and Use Committee (Temple, TX, USA). Male Yorkshire pigs, aged 8–12 weeks old and weighing 8–11 kg, were obtained from Real Farms (San Antonio, TX, USA). The cytotoxic glucose analog streptozocin (STZ; Zanosar^®^, 200 mg/kg in saline; Teva Parenteral Medicines, Irvine, CA, USA) was given intravenously via an ear vein (37 pigs) as described in detail in our previous studies to develop type 1 diabetes [71,72]. In the control group, saline was given intravenously instead of STZ (32 pigs). The pigs were nurtured for a period of 2 weeks. All animals were granted free access to water and fed with glucose syrup mixed with hog chow to prevent a sudden drop in blood sugar, especially during the first 24 h after STZ injection. The pigs were allowed free access to water and commercial pig chow afterwards. The general condition, body weight, and the level of blood glucose assessed with a Bayer Contour glucometer (Bayer HealthCare, Mishawaka, IN, USA) were recorded in all pigs.

The pigs that developed sustained hyperglycemia with a fasting blood glucose level between 250 and 600 mg/dL were included as a diabetic group in the present study. Pigs with sustained blood glucose above 600 mg/dL were treated with insulin (Humulin^®^ 70/30, 2–8 units; Lilly; Indianapolis, IN, USA) to keep the glucose level between 250 and 600 mg/dL. Insulin was not given within 48 h before the terminal surgery. After the 2-week period, the pigs were sedated with Telazol (4–8 mg/kg intramuscularly; TW Medical Veterinary Supply, Austin, TX, USA), gas anesthetized with 2% to 5% isoflurane (Baxter Healthcare Co., Deerfield, IL, USA), heparinized with heparin (1000 U/kg, intravenously via marginal ear vein; Cardinal Health, Dublin, OH, USA), and intubated. The heart was excised and immediately kept on iced (5 °C) saline.

### 4.2. Isolation and Cannulation of Coronary Arterioles

Microscopic dissection of individual subepicardial arterioles (≈1 mm in length; ~20–60 µm in situ diameter) from the surrounding cardiac tissue was performed [41]. The coronary arterioles were then cannulated and ligated on each end with glass micropipettes containing a physiological saline solution (PSS)-albumin (1%; Thermo Fisher Scientific, Bridgewater, NJ, USA). The coronary arterioles were then pressurized to 60 cmH_2_O intraluminal pressure without flow by two independent pressure reservoir systems [12]. The inner diameter of coronary arterioles was continuously monitored using videomicroscopic techniques throughout the experiments, as described previously [12,41].

### 4.3. Study of Vasomotor Function

The cannulated coronary arterioles were bathed in physiological saline solution with albumin at 36 to 37 °C to allow the development of basal tone (stable within 60 min). To evaluate the effect of diabetes on vasomotor function, flow-induced vasodilation to a step-wise increase in pressure gradient (from 0 to 60 cmH_2_O) and concentration-dependent vasodilations to VEGF (1 pmol/L to 1 nmol/L) [20,73] and sodium nitroprusside (10 nmol/L to 10 µmol/L) [74] were recorded in vessels isolated from the 2-week diabetic and saline control pigs. To assess the involvement of uncoupled eNOS in the diabetes-induced effect, vasodilation was examined after treating the vessels with L-NAME (10 μmol/L) [50], catalase (1000 U/mL) [10], or L-arginine (3 mmol/L) [75] for 30 min. The role of vascular arginase in flow-induced dilation was assessed after treating the vessels with arginase inhibitor nor-NOHA (0.1 mmol/L; Cayman Chemical, Ann Arbor, MI, USA) [10,61]. The impact of PI3K on the VEGF- and flow-induced vasodilations was evaluated after incubation with PI3K inhibitor wortmannin (0.1 µmol/L) for 30 min [20].

### 4.4. RNA Isolation and Reverse Transcription Polymerase Chain Reaction (RT-PCR) Analysis of Antioxidant Transcripts

Total RNA was isolated from the coronary arterioles (3–4 vessels per sample pooled from one heart) of similar size to those used for functional studies, as we described previously [57]. Sets of primers specific for SOD1 (sense: 5′-CAGGTCCTCACTTCAATCC-3′, antisense: 5′-CCAAACGACTTCCASCAT-3′) (Accession no.NM_001190422.1), catalase (sense: 5′-CGAAGGCGAAGGTGTTTG-3′, antisense: 5′-AGTGTGCGATCCATATCC-3′) (Accession no. NM_214301.2), and glutathione peroxidase (GPX, sense: 5′-CACAACGGTGCGGGACTA-3′, antisense: 5′-CATTGCGACACACTGGAGAC-3′) (Accession no. NM_214201.1) genes were engineered (Sigma-Genosys, The Woodlands, TX, USA). Using 0.5 µg of total RNA per sample, RT-PCR was conducted, as we described previously [57]. The PCR reaction was optimized and run for 35 cycles for all genes. The level of expression of SOD1, catalase, and GPX transcripts was normalized to that of 18S transcripts (sense: 5′-GCGGCTTTGGTGACTCTA-3′, antisense: 5′-CTGCCTCCTTGGATGTG-3′) (Accession no. NR_002170.3).

### 4.5. Chemicals

All drugs were obtained from Sigma-Aldrich (St. Louis, MO, USA) except as specifically stated. Wortmannin was dissolved at 10 mmol/L in dimethyl sulfoxide, with subsequent concentrations diluted in PSS. All other drugs were dissolved in PSS. The final concentration of dimethyl sulfoxide in the vessel bath did not exceed 0.03% by volume. The 0.03% dimethyl sulfoxide had no significant effect on vessel viability, vasodilator responses, or maintenance of tone as previously reported [76].

### 4.6. Data Analysis

At the end of each experiment, the changes in the arteriolar diameter in response to the pressure gradient (flow-induced dilation) or vasoactive agonists were normalized to the maximum diameter changes in response to 0.1 mmol/L sodium nitroprusside in the presence of calcium-free PSS with EDTA (1 mmol/L) [77]. Diameter changes were expressed as % of maximum dilation. Data are reported as mean ± SEM and *n* value describes the number of vessels studied (one per pig per treatment group) in functional analysis or the number of pigs used in molecular studies. Student’s *t*-test or ANOVA followed by the Bonferroni multiple range test was used to determine the significance of experimental interventions, as appropriate. A value of *p* < 0.05 was considered significant.

## Data Availability

The datasets generated and/or analyzed for the current study are available from the corresponding authors upon reasonable request.
